# An Improved Performance Frequency Estimation Algorithm for Passive Wireless SAW Resonant Sensors

**DOI:** 10.3390/s141222261

**Published:** 2014-11-25

**Authors:** Boquan Liu, Chenrui Zhang, Xiaojun Ji, Jing Chen, Tao Han

**Affiliations:** School of Electronic Information and Electronic Engineering, Shanghai Jiao Tong University, Shanghai 200240, China; E-Mails: laubor@yeah.net (B.L.); gmancn@163.com (C.Z.); jxj127@sjtu.edu.cn (X.J.); jingchen0408@hotmail.com (J.C.)

**Keywords:** wireless SAW sensors, singular value decomposition, frequency estimation

## Abstract

Passive wireless surface acoustic wave (SAW) resonant sensors are suitable for applications in harsh environments. The traditional SAW resonant sensor system requires, however, Fourier transformation (FT) which has a resolution restriction and decreases the accuracy. In order to improve the accuracy and resolution of the measurement, the singular value decomposition (SVD)-based frequency estimation algorithm is applied for wireless SAW resonant sensor responses, which is a combination of a single tone undamped and damped sinusoid signal with the same frequency. Compared with the FT algorithm, the accuracy and the resolution of the method used in the self-developed wireless SAW resonant sensor system are validated.

## 1. Introduction

Resolution is one of the most important parameters of passive wireless SAW sensors [1–4]. Among all wireless SAW sensor configurations, a resonant sensor can potentially achieve better theoretical resolution (with the assumption of infinite signal-to-noise (SNR)) with a significant reduction of SAW chip size. In practical, the resolution is mainly determined by noise in the measured value, which in its turn depends on the loaded quality factor (*Q* factor) and the insertion losses of the SAW resonator, noise properties of the receiver and the algorithm used for spectrum estimation. So far, studies on the influences of the loaded *Q* factor and the additive white Gaussian noise on the standard deviation of the resonant frequency measured by means of pulsed interrogation of the SAW resonator were performed [5–7].

In most published papers on wireless SAW resonant sensors, Fourier transform (FT) calculations are still commonly used for resonance frequency estimation [5–9]. Even though the interpolation leads to a performance improvement, in the case of long distance and low *Q* factor, the length of the effective response signal would be reduced, resulting in frequency estimation errors in the traditional SAW resonant sensor system due to the resolution restriction of the FT approach.

The use of model-based parametric methods for SAW sensor frequency estimation was mentioned in [2]. Indeed, many model-based parametric methods have been continually proposed in the field of signal processing to overcome the defects of FT. However, due to the application of the limiting amplifier in the sensor reading system, the received response consists of the partial undamped and the damped hybrid sinusoid signals. The performance of parametric methods in practical SAW resonator systems has not been clearly documented. We find that some parametric methods like auto-regressive and moving average (ARMA), and the Pisarenko harmonic decomposition method, although theoretically they provide better resolution than the FT method for short data, most of them become prone to errors for the non-stationary damped sinusoidal signals and even worse than the FT method at low signal-to-noise ratio (SNR) [10–12]. Most of the computationally attractive schemes such as Prony, iterative filtering and weighted phase average (WPA) approaches, have the demerits of poor threshold performance, non-uniform estimation performance across the admissible frequency range and limited frequency operation range [13]. The conventional singular value decomposition (CSVD)-based parametric methods such as Kumaresan-Tufts (KT) and matrix pencil approaches can obtain accurate estimations when the SNR is above 5 dB [14,15]. However, signal modeling using a large scale Hankel-style matrix leads to a large-sized polynomial which becomes a computational bottleneck in solving the linear prediction equations. Moreover, noise introduces extra perturbations to the extraneous roots of large-sized polynomials when the SNR is reduced to a certain degree, resulting in accuracy loss [10].

Recently, a singular value decomposition-based single frequency estimation method has attracted much attention due to its high computational efficiency and good performance [16,17]. Its computational complexity is significantly reduced by constructing a signal matrix with no repeated entries; its threshold SNR is further reduced to about −5 dB. In this paper, we extend this approach to amplitude hybrid sinusoid signals. The performance of this algorithm on different amplitude combinations of the damped and the undamped parts in the hybrid signal is investigated.

The rest of this paper is organized as follows: in Section 2, we describe the operating principle of passive wireless SAW resonant sensor measurement system and the used frequency measurement approach. Simulation results are presented in Section 3, and its practical experimental results are introduced in Section 4. Finally, the conclusions are given in Section 5.

## 2. Frequency Measurement Algorithm

### 2.1. The Principle of Passive Wireless SAW Resonant Sensor System

**Figure 1.**
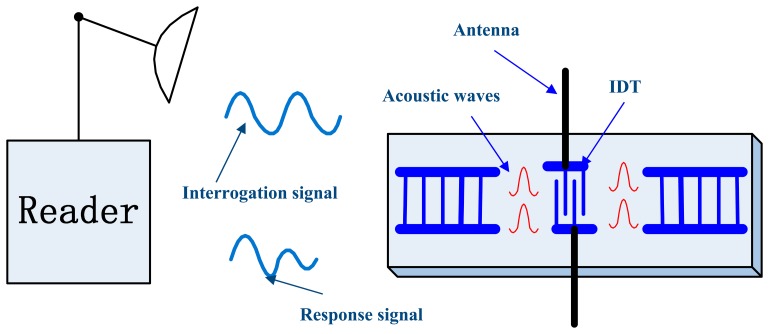
Passive wireless SAW resonant sensor system block diagram.

The operation principle of a passive wireless SAW resonant sensor system is illustrated in Figure 1. The interrogation unit emits a high frequency electromagnetic wave (radio frequency (RF) interrogation signal) to activate a passive SAW resonant sensor, which consists of a piezoelectric substrate and a one-port SAW resonator. The interdigital transducer (IDT), which is connected to the antenna, , transforms the received electromagnetic wave into a narrow-band SAW with the help of the inverse piezoelectric effect. The practical resonant frequency depends on the structure of the resonating cavity and the environmental influences the resonator is exposed to. After the stimulating signal is switched off, the in-band various frequency components of the SAW oscillate freely damped with different time constants. The SAW returns to the antenna for retransmission to the interrogation unit as an electromagnetic wave with the help of IDT. The longest echo duration is achieved only when the frequency of the emitted electromagnetic wave is equal to the resonance frequency of SAW resonator. The interrogation unit evaluates the frequency of this damped electromagnetic wave and determines the value of the measurand.

### 2.2. Signal Model

The received SAW resonator response signal is an amplitude combination of a single tone undamped and damped sinusoid signal with the same frequency, it can be recorded as:

(1) $$\begin{array}{cc} x_{k}=s_{k}+{\tilde{n}}_{k}, & k=1,2,\ldots ,\text{K} \end{array}$$

where:

(2) $$s_{k}=Ae^{-(dk)}\sin(\omega k+\phi )$$

the *d* = 0 when 1 < *k* ≤ *k_d_*, when *k* > *k_d_* the damping happens, *d* = (*πf_n_*) / *Q*. The *f_n_* is the frequency of natural oscillation of the resonant SAW sensor. The *A*, *ω* ∈ (0, *π*) and *ϕ* ∈ (0, 2*π*) are the amplitude, frequency and phase of received resonator response signal *s_k_*, respectively, and they are unknown constants, while *ñ_k_* is the noise which is injected in the transmission channel or produced by signal conditioning in the measurement device with unknown variance of *σ*^2^.

Unlike a Hankel-style matrix in the conventional SVD based methods, a signal data matrix with no repeated entry is constructed for the used method as shown in Equation (3):

(3) $$X=S+N=\left[\begin{array}{cccc} x_{1} & x_{M+1} & \cdots & x_{(M-1)N+1}\\ x_{2} & x_{M+2} & \cdots & x_{(M-1)N+2}\\ \vdots & \vdots & \vdots & \vdots \\ x_{M} & x_{2M} & \cdots & x_{MN} \end{array}\right]$$

where ***S*** is the noiseless signal matrix containing {*s_k_*} and *MN* = *K*. *K* is the number of samples in {*x_k_*} depending on sampling frequency and time, and if it is not factorizable, one simple way is to discard a few samples and find *M* and *N* such that their product is closest to *K*. In practical, *N* is chosen as close as possible to *M* so as to improve computational complexity.

### 2.3. The Algorithm Principles

Based on the fact that the rank of a single frequency noiseless signal matrix ***S*** is two, the optimum estimation of ***S*** is taken as the product of two largest singular values *λ*_1_, *λ*_2_ and their left and right singular vectors ***u***_1_, ***u***_2_ and ***v***_1_, ***v***_2_ of noisy received signal matrix ***X*** corresponding to the signal subspace [16]. Therefore, by taking advantage of partial singular value decomposition, the noiseless response signal matrix ***S*** can be approximated by:

(4) $$\hat{S}=[{\text{u}}_{1}{\text{u}}_{2}]\text{diag}(\lambda_{1},\lambda_{2}){\left[{\text{v}}_{1}{\text{v}}_{2}\right]}^{T}$$

where ***u***_1_, ***u***_2_ and ***v***_1_, ***v***_2_ are unit vectors. Since other singular vectors corresponding to the noise subspace will no longer affect the solution, the noise sensitivity of algorithm is reduced.

According to the trigonometric identity, [***v****_i_*]*_n_* + [***v****_i_*]*_n_*_−2_ = 2cos(*Mω*) [***v****_i_*]*_n_*_−1_, which can be satisfied even for the damped data when the sampling rate is high enough. In the case of frequency estimation, compared with the left singular vectors, the frequency calculated from right singular vectors leads to a smaller variance [17–19]. Therefore, the resonant frequency can be firstly estimated from ***v***_1_, ***v***_2_. In the algorithm, the weighted least squares (WLS) technique can be used for improving performance [20]. There is:

(5) $$\cos(M\omega )=\frac{{\text{y}}^{T}W_{\text{v}}\text{z}}{2({\text{y}}^{T}W_{\text{v}}\text{y})}$$

where ***W****_v_* is the optimal weighting matrix and can be computed as:

(6) $${\text{W}}_{v}=\text{diag}(\lambda_{1}^{2},\lambda_{2}^{2})⊗{\left({\text{BB}}^{T}\right)}^{-1}$$

with $\text{B}=\text{Toeplitz}({\left[1\;0_{1\times (N-3)}\right]}^{T},[1-\frac{{\text{y}}^{T}{\text{W}}_{v}\text{z}}{\left({\text{y}}^{T}{\text{W}}_{v}\text{y}\right)}1\;0_{1\times (N-3)}])$, $\text{y}={\left[{\text{y}}_{1}^{T}y_{2}^{T}\right]}^{T}$, $\text{z}={\left[{\text{z}}_{1}^{T}z_{2}^{T}\right]}^{T}$, ***y****_i_* = [[***v****_i_*]_2_ [***v****_i_*]_3_⋯[***v****_i_*] *_N_*] and ***z****_i_* = [[***v****_i_*]_1_ [***v****_i_*]_3_ [***v****_i_*]_2_ + [***v****_i_*]_4_⋯ [***v****_i_*]*_N_*_−2_ +[***v****_i_*]*_N_*].

The frequency estimations calculated from the right singular vectors are denoted by *ω_v_*,*_j_*, *j* = 1,2, …*M*:

(7) $$\omega_{v,j}=\frac{1}{M}[{\left(-1\right)}^{j}\arccos(\frac{{\text{y}}^{T}{\text{W}}_{v}\text{z}}{2({\text{y}}^{T}{\text{W}}_{v}\text{y})})+\left\lfloor \frac{j}{2}\right\rfloor 2\pi ]$$

Note that the *ω_v_*,*_j_* corresponds to *M* of possible frequency estimations, ⌊ ⌋ rounds the value to the nearest integer towards −∞. Since the reflective echo is composed of a single frequency signal, similar to the above procedure, a frequency estimation of *ω_u_* should be implemented in terms of the left singular vectors ***u***_1_ and ***u***_2_:

(8) $$\omega_{u}=\arccos(\frac{{\text{p}}^{T}{\text{W}}_{u}\text{q}}{2({\text{p}}^{T}{\text{W}}_{u}\text{q})})$$

where:

(9) $${\text{W}}_{u}=\text{diag}(\lambda_{1}^{2},\lambda_{2}^{2})⊗{\left({\text{CC}}^{T}\right)}^{-1}$$

with $\text{c}=\text{Toeplitz}({\left[1\;0_{1\times (M-3)}\right]}^{T},[1-\frac{{\text{p}}^{T}{\text{W}}_{u}\text{q}}{\left({\text{p}}^{T}{\text{W}}_{u}\text{q}\right)}1\;0_{1\times (M-3)}])$, $\text{p}={\left[{\text{p}}_{1}^{T}{\text{p}}_{2}^{T}\right]}^{T}$, $\text{q}={\left[{\text{q}}_{1}^{T}{\text{q}}_{2}^{T}\right]}^{T}$, ***p****_i_* = [[***u****_i_*]_2_[***u****_i_*]_3_⋯[***u****_i_*]*_M_*] and ***q****_i_* = [[***u****_i_*]_1_ + [***u****_i_*]_3_ [***u****_i_*]_2_ + [***u****_i_*]_4_⋯ [***u****_i_*]*_M_*_−2_ +[***u****_i_*]*_M_*].

Here, though with less accuracy, *ω_u_* is the sole frequency estimation from Equation (8). Thus, combining *ω_u_* and {*ω_v,j_*}, the optimum estimation *ω̂* can be determined:

(10) $$\hat{\omega }=\arg\min_{\omega_{v,j},j\in \{1,2,\ldots M\}}\left|\omega_{v,j}-\omega_{u}\right|$$

## 3. Simulation Results

In the simulations, the signal frequency is 500 kHz and the sampling frequency *f_s_* is set to 9 Msps, which is similar to the intermediate frequency and the sampling frequency in the reading transceiver. To verify the accuracy of the measurement algorithm, the performance of the used algorithm on different combinations of damped and undamped parties compared with the commonly used FFT. The complex sinusoidal signals with the same frequency are used for FT; and the length of each signal is extended to 8192 by using zero padding before performing FT; the estimated frequency of FT method is obtained by a parabolic approximation using the maximum of spectrum amplitude and its two adjacent points [21]. The used noises are zero-mean white Gaussian noises. The SNR of this hybrid signal is defined as:

(11) $$\begin{array}{cc} \text{SNR}=10\log_{10}\frac{\overset{K}{\underset{k=1}{\text{∑}}}{\left|s_{k}\right|}^{2}}{\overset{K}{\underset{k=1}{\text{∑}}}{\left|{\tilde{n}}_{k}\right|}^{2}} & k=1,2,\ldots ,K \end{array}$$

the |·| means the absolute operator.

All the results are averages of 1000 Monte Carlo experiments. The mean square error (MSE) of the estimated frequency and actual value is calculated as following:

(12) $$\text{MSE}=10\log_{10}{\left(\frac{\hat{\omega }-\omega }{2\times \pi }\right)}^{2}$$

### 3.1. Accuracy of Measurement

**Figure 2.**
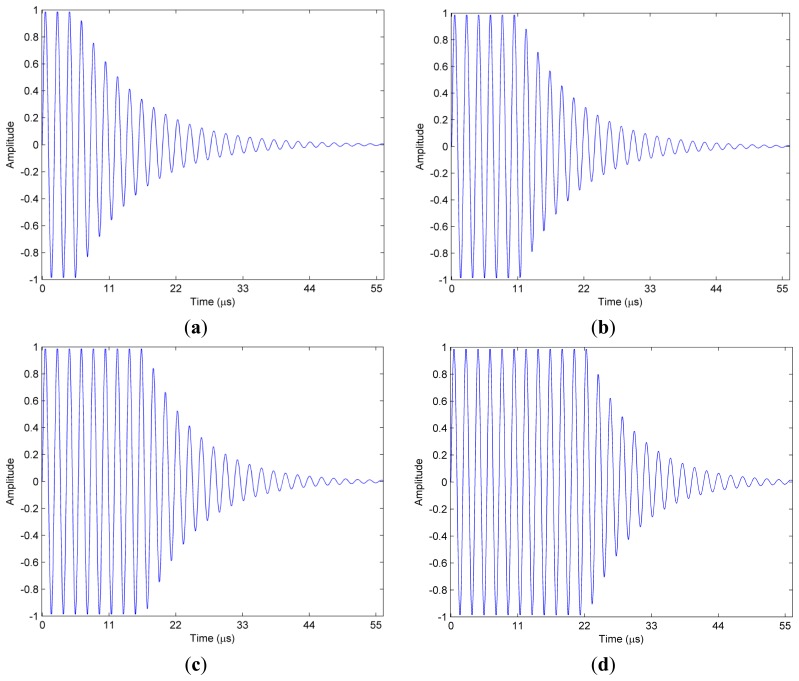
Hybrid signals with different undamped part ratio (**a**) 10% (**b**) 20% (**c**) 30% (**d**) 40%.

**Figure 3.**
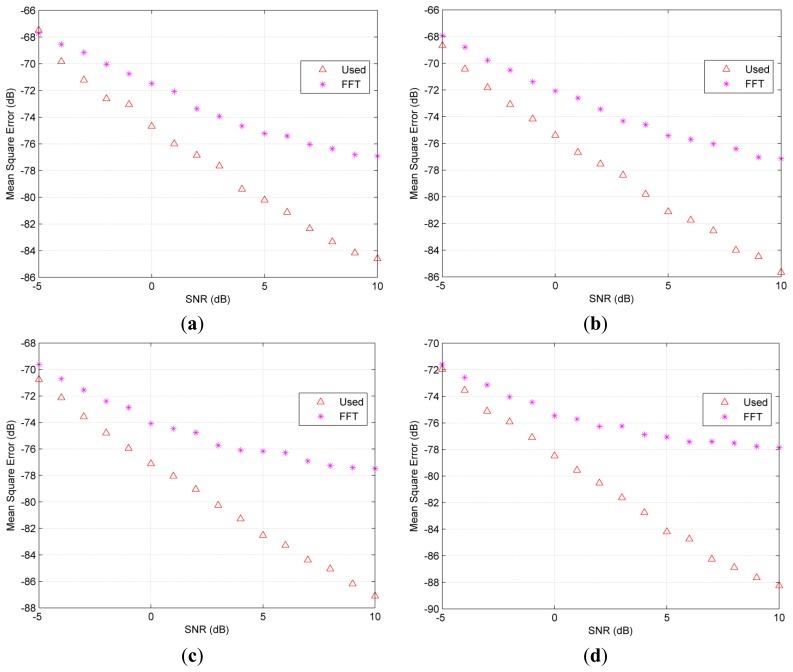
Comparisons of accuracy on mean square frequency error versus SNR on different undamped ratios (**a**) 10% (**b**) 20% (**c**) 30% (**d**) 40%.

The hybrid signals with the effective time lengths of 56 μs used in the simulation are shown in Figure 2. The comparisons between the used algorithm and FFT in the different combinations of undamped and damped parts are shown in Figure 3. As demonstrated, the MSE of the FFT are above the used method if the undamped ratio is bigger than 10% or the SNR is above −4 dB. The undamped ratio means the time length of the undamped part to the whole effective time length. Furthermore, both a bigger undamped ratio and a higher SNR lead to a wider MSE gap between the used method and FFT. FT method suffers from the biased frequency estimation due to the non-integer periodical truncation. Thus, the MSE presented in Figure 3 for the FFT method does not drop with SNR at the same rate when SNR is bigger than 7 dB.

### 3.2. Resolution of Measurement

Since the resolvability of two frequencies depend on the SNR and the *Q* factor of the resonator, we consider the accuracy of the results over various *Q* factors at the given SNR. Here, the time lengths of undamped parts of the hybrid signals are all 14 μs. Since the amplitude of exponentially damped part is dependent on *e*^−(^*^π^*^×^*^f_n_^*^)/^*^Q^*, the bigger *Q* factor represents the longer effective time length.

**Figure 4.**
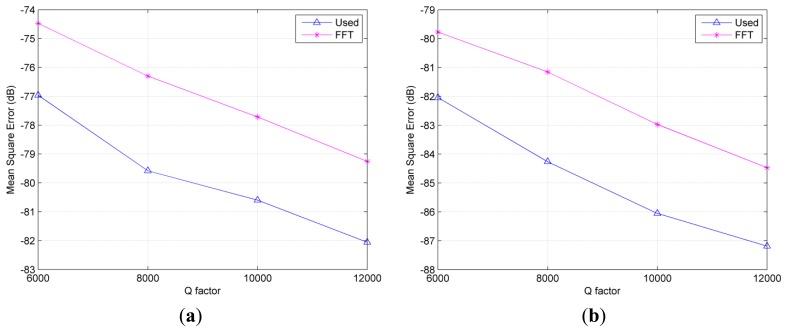
Comparisons of measurement resolution at different SNR levels. (**a**) SNR level = 5 dB (**b**) SNR level = 10 dB.

In order to evaluate the resolvability of the algorithms, the average frequency estimation of 1000 times rather than the real value of *ω* is specially used in Equation (12). The corresponding results for the used method and the FFT technique at different SNR levels are presented in Figure 4. It can be seen that the used algorithm exhibits smaller values of MSE than FFT method even in the short effective time length. However, for the conventional SVD based method, such as the KT algorithm, its variance is higher than the FFT method with parabolic interpolation if the signal is not too long [22]. That is, the used method shows better resolution ability. Furthermore, it indicates a longer sensing distance than using the FT method.

## 4. Experimental Results

**Figure 5.**
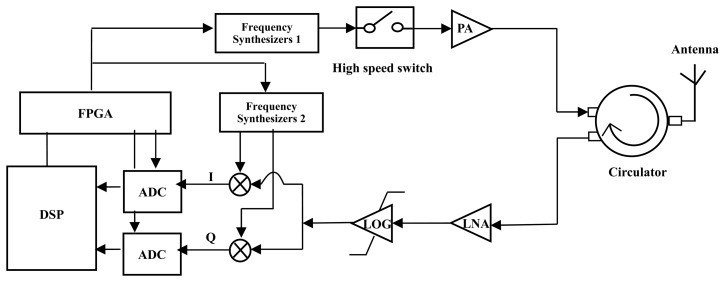
Block diagram of the interrogation unit.

The Figure 5 depicts the block diagram of the transceiver unit. The power amplifier (PA) boots the transmitted power up to 27 dBm. The frequency difference between the transmitter and in the receiver is digitally controlled at fixed 500 kHz. The phase noise of the frequency synthesizers are within −110 dBc/Hz at 100 kHz offset. The amplifier (LOG) is used to provide an undamped output when the input signal is beyond −70 dBm due to impedance mismatching (the theoretical value is −78 dBm). When the input power of receiver unit is −98 dBm, the SNR of the signal offered to the analog-to-digital converters (ADC) is about 10 dB. The reflected echoes are converted into the digital signals by using 14 bit ADC with sampling rate of 9 Msps. The recorded data are processed using the proposed algorithm and FFT, respectively. The time sequence of the interrogation unit is well controlled so as to keep the initial phase angle of the interrogation as a constant during all the measurement processing. In the case of the *Q* factor is 6000 and the sensing distance is 2 m, the time length of the undamped part can be guaranteed above 15 μs. That is, the undamped part ratio in the received signal is at least 26%.

**Figure 6.**
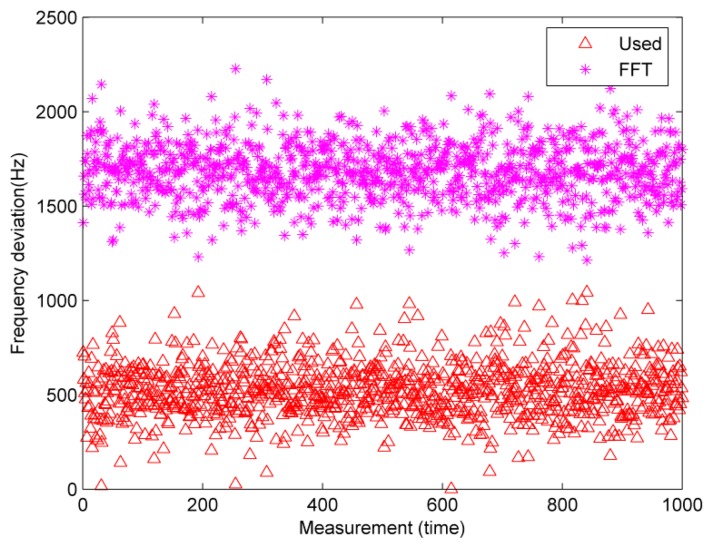
Absolute values of the deviations from the actual frequency.

The accuracy of the used algorithm is firstly verified by an experiment, which employs a signal generator (IFR 2023A) to simulate the response signal. The transmitted power is set to −79 dBm, which is higher than the sensitivity of the practical receiver. The absolute values of the deviations from the actual frequency are shown in Figure 6. It demonstrates that the results of the used method are much closer to the actual frequency, that is, the used method has higher accuracy. The average deviations are 1.67 KHz and 529.89 Hz for the FFT and used method, respectively.

**Figure 7.**
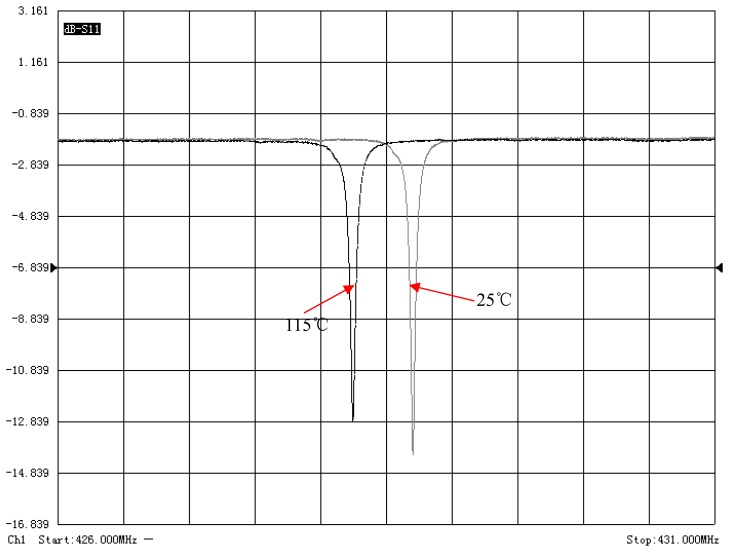
The measured reflection coefficient (S_11_) of a resonator at 25 °C and 115 °C, respectively.

**Figure 8.**
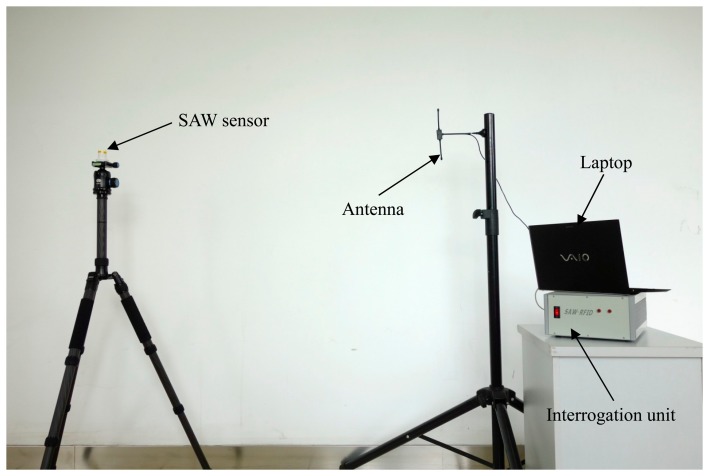
Experimental arrangement.

The algorithm is also validated using the actual wireless SAW resonating sensor which is made of Y-rotated 36° (AT-cut) quartz. Its thermal sensitivity can be accurately adjusted by controlling the metallization thickness (*h/λ*) of the SAW resonator. In our experiment, the metallization thickness of 4% is designed; thus the temperature-induced frequency shift of SAW resonators can be within 950 kHz in the range from −40 °C to +150 °C, *i.e.*, the average temperature sensitivity of the sensor is about 4.9 KHz/°C. The measured S_11_ frequency responses of the SAW resonator using the vector network analyzer AV3629A are shown in Figure 7. Two curves are measured at 25 °C and at 115 °C, respectively. And the central frequency of the resonator shifts from 428.689 MHz to 428.253 MHz, which is in good agreement with the designed temperature sensitivity and the loaded *Q* factor also slightly decreases from 6679 to 6397. As shown in Figure 8, in this experiment, the resonant SAW sensor and the antenna are mounted on two separate tripods to keep the reading distance of 2 m.

**Figure 9.**
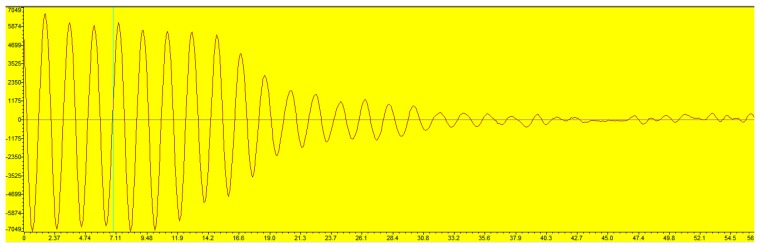
SAW resonator response signal (the horizontal axis is time in microsecond, and the vertical axis is the digitalized amplitude).

**Figure 10.**
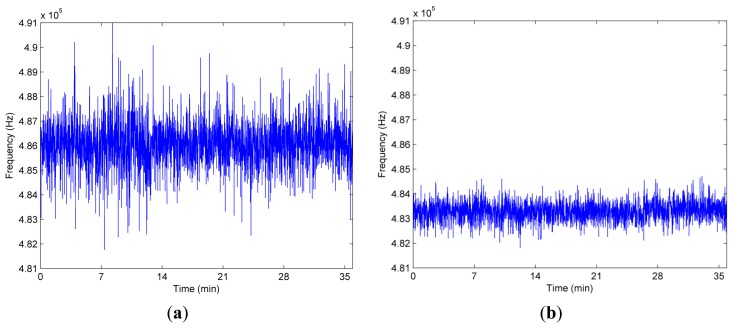
Measured frequency over the measurement time. (**a**) FFT; (**b**) Used method.

An acquisition of SAW resonator response in the digital signal processor is shown in Figure 9. The comparison of the measured resonant frequencies between the proposed SVD based system and the FFT based conventional system over measurement time in a fixed temperature is shown in Figure 10. Enhanced performance in terms of reduced variance is visible. More specifically, the standard deviation of the used method is 391.26 Hz at the room temperature. Using the same measured data, however, the corresponding standard deviation of the FFT scheme is 951.05 Hz. Clearly, in the same condition, the standard deviation of used algorithm is up to a factor 2.43 lower than the FFT based system. Thus, the proposed system delivers lesser standard deviation, that is, higher resolution than the conventional system using FFT.

## 5. Conclusions

An efficient frequency estimation method considering a single frequency measurement requirement is used to estimate the echo frequency of SAW resonant sensors. The theoretical properties, simulations and practical experiments demonstrate that the self-developed system using this SVD based method shows some properties in high computational efficiency, high accuracy and resolution. It has been found that the algorithm can achieve better resolution and accuracy than the FT method as long as the ratio of the undamped part in the hybrid signal exceeds a certain value. Thus, using a logarithmic amplifier in the self-developed wireless transceiver, better measurement accuracy and longer reading distance are verified by simulation and experiment.
